# Meeting the support and information needs of women with advanced breast cancer: a randomised controlled trial

**DOI:** 10.1038/sj.bjc.6603320

**Published:** 2006-09-12

**Authors:** S Aranda, P Schofield, L Weih, D Milne, P Yates, R Faulkner

**Affiliations:** 1 Peter MacCallum Cancer Centre, St Andrews Place, Locked Bag 1, A'Beckett Street, East Melbourne, Victoria 8006, Australia; 2 School of Nursing, University of Melbourne, Parkville, Victoria, Australia; 3 Faculty of Medicine, Dentistry and Health Sciences, University of Melbourne, Parkville, Victoria, Australia; 4 School of Nursing, Queensland University of Technology, Brisbane, Queensland, Australia

**Keywords:** advanced breast cancer, intervention, patient, perceived needs, quality of life, supportive care

## Abstract

Addressing psychosocial and quality of life needs is central to provision of excellent care for people with advanced cancer. This study tested a brief nurse-delivered intervention to address the needs of urban women with advanced breast cancer. This study was conducted at four large urban hospitals in Australia. One hundred and five women with advanced breast cancer were recruited and randomised to receive the intervention or usual care, then asked to complete the European Organisation of Research and Treatment of Quality of life Q-C30 version (2.0) (EORTC Q-C30) (version 2) and Supportive Care Needs Survey (SCNS) at 1 month and 3 months postrecruitment. No significant differences were detected between intervention and usual care groups in the SCNS or the EORTC Q-C30 subscale scores. However, when the groups were divided into high needs (score of above 50) and low baseline needs (score of 50 or below) for each SCNS subscale, a significant difference between intervention and usual care groups was found in the psychological/emotional subscale among women with high baseline needs. In conclusions, this study demonstrated that a face-to-face session and follow-up phone call with a breast care nurse significantly reduced the psychological and emotional needs of those with high initial needs. There was no evidence of the intervention influencing the quality of life; or perceived needs of women with low initial psychological/emotional needs or perceived needs in other domains. Possibly, the intervention was not sufficiently intense to achieve an effect.

Advanced breast cancer is the most common cause of cancer death in women from developed countries (Stewart and Kleihues, 2003). When a person has incurable disease, optimising quality of life and meeting the woman's psychosocial and information needs must be central to excellent care (Aranda *et al*, 2005). Extending survival time with aggressive treatments without considering these issues may do more harm than good. It is critical to address the substantial psychosocial and informational needs of people with advanced cancer (Sanson-Fisher *et al*, 2000; Aranda *et al*, 2005). This study reports a trial of a brief, structured, nurse-delivered intervention designed to meet the psychosocial, informational and self-care needs of women with advanced breast cancer.

## 

### Previous research on psycho-educational interventions with people with cancer

In a systematic review (Newell *et al*, 2002) of psychological interventions for cancer patients, it was concluded that a range of interventions including group-based and individual therapies, informational and educational interventions, guided imagery and cognitive behavioural therapies showed promise for improving patient outcomes and warranted further rigorous research.

Many studies involving psycho-educational interventions for women with breast cancer have demonstrated positive results; however, the majority of them were delivered by a trained psychologist or psychiatrist (Newell *et al*, 2002). Another recent systematic review of psychological group interventions for women with metastatic breast cancer concluded that there was evidence that group therapies, either cognitive-behavioural or support-expressive, produced short-term psychological improvements (Edwards *et al*, 2004). However, in each of the trials reviewed, the intervention was delivered by a trained therapist and involved a series of sessions.

To date, there has been limited research on the impact of nurse-delivered psycho-educational interventions for people with cancer, but the trials that have been conducted have been mostly with early breast cancer patients. A large, well-designed randomised trial showed that a breast care nurse providing empathic encouragement of emotional expression, combined with individualised information provision, reduced psychological morbidity (McArdle *et al*, 1996). Another earlier trial demonstrated that a regular contact with a specialist breast care nurse improved psychosocial outcomes postmastectomy including social recovery, returning to work, and adaptation to breast loss (Maguire *et al*, 1983). Cognitive behavioural techniques are known to be effective in addressing issues faced by breast cancer patients. For example, a recent trial demonstrated that cognitive behavioural techniques were effective in reducing insomnia among breast cancer patients; however, this was administered by a psychologist (Savard *et al*, 2005). Another randomised controlled trial demonstrated that cancer-related fatigue in women with breast cancer is significantly reduced by a three individualised, educational and support sessions, which drew on cognitive behavioural techniques and was delivered by a specialist nurse (Yates *et al*, 2005). Other recent studies with breast cancer patients have shown mixed or negative results. (Edelman *et al*, 1999; Edmonds *et al*, 1999; Scholten *et al*, 2001; Owen *et al*, 2005). However, none of these trials used a specialised breast nurse. Moreover, none *systematically* tailored the intervention to each patient's baseline specific needs and this may explain the negative findings. Further few studies have been conducted on nurse-led interventions in the context of advanced breast cancer. Use of the breast nurse in provision of psychoeducational support to women with advanced breast cancer offers the possibility of routine implementation of such support into practice and as such warrants investigation.

### The present study

Effective intervention involves addressing psychological needs and providing relevant information and self-care strategies to address the patient's unique needs, including symptom management (Schofield *et al*, in press). Previous successful psychoeducational interventions tended to be intense, involving many face-to-face interactions, but not tailored to the individuals' needs (Newell *et al*, 2002). Psychoeducational interventions typically involve a minimum of six contact hours with some entailing over 100 h of therapy (Newell *et al*, 2002). Unfortunately, such resource intensive psychoeducational interventions are unlikely to be disseminated into routine clinical practice (Schofield *et al*, in press). Hence, the very brief intervention developed for this study was designed to be more targeted than its forerunners, because it used a systematic mechanism to assess and meet the woman's needs. Very few trials have investigated brief interventions, and those that have tended not to produce significant change, probably due to the lack of *systematic* individual tailoring (Newell *et al*, 2002). One exception was a study by Velikova *et al* (2004). This study showed that systematically assessing patients' quality of life and providing this information to the treating doctor improved patient quality of life and emotional functioning.

The content of the intervention was designed around the FOCUS framework (Northouse *et al*, 2002). FOCUS stands for Family involvement, Optimistic attitude, Coping effectiveness, Uncertainty reduction and Symptom management. Family members were included in the intervention because the involvement of others in psychosocial interventions for cancer patients reduces significantly patient anxiety and distress (Newell *et al*, 2002). An intensive five-session intervention using this framework and delivered by a specialist nurse has been found to be effective for women with advanced breast cancer in terms of reducing feelings of hopelessness (Northouse *et al*, 2005). In this study, cognitive-behavioural techniques were used to improve coping, levels of optimism and self-care management. Adult learning principles were also adopted (Knowles, 1989). In particular, education was centred on the woman's salient and immediate concerns. Also, learning occurred in a quiet and comfortable environment. The relevant information presented was individualised and delivered in manageable amounts. Recall of key information was enhanced by repetition (Ley and Llewellyn, 1993).

*Aim*: The aim was to examine the effectiveness of a brief, nurse-delivered intervention designed to address the individual needs of women with advanced breast cancer. The goals of the intervention were to elicit women's key concerns; to provide relevant information; to promote self-care activities in relation to symptom management and to refer women to appropriate support.

*Hypotheses*: Those patients randomly allocated to the intervention group will report a decrease in psychological and informational needs and an increase in quality of life from baseline to follow-up compared to women receiving usual care.

## MATERIALS AND METHODS

### Setting

This study was conducted at the outpatient clinics of four large, urban hospitals, three public and one private, in Melbourne, Australia.

### Sample

A consecutive sample of 172 women was approached. Inclusion criteria for patients were as follows: a diagnosis of breast cancer that was newly diagnosed at an advanced stage, recurred or progressed in the preceding 12 months; aged 18 years or older; had sufficient English for the study requirements; and had access to a telephone for follow-up. Of these, 67 declined to participate (a consent rate of 61%). All those who consented completed the baseline questionnaire (*n*=105).

### Design and procedure

Ethics committee approval was obtained from all study sites. The design was a two-group randomized-controlled trial with data collected at baseline (time 1) then after the intervention at one month (time 2) and 3 months (time 3) postbaseline.

#### Recruitment

The research breast care nurse (BCN-R) identified potentially eligible women. After their treating doctor had provided initial information about the study, the BCN-R obtained verbal and written consent. The woman was then asked to complete the baseline questionnaire. The baseline questionnaire contained items of demographic data, the Supportive Care Needs Survey (SCNS) (Bonevski *et al*, 2000) and the European Organisation of Research and Treatment of Cancer Quality of Life Q-C30 version (2.0) (EORTC QLQ-30) (Kaasa *et al*, 1995). Diagnostic and treatment details for each patient were obtained from the patient's medical record.

#### Randomisation

For each study site, an even number of folded intervention (20) and control (20) cards were thoroughly shuffled then placed in consecutively numbered, opaque, sealed envelopes. After baseline data collection, an envelope was sequentially drawn and opened by the BCN-R.

#### Control group

Women in the control group received the standard care given at the treatment site, including referral to breast care nurse or cancer support nurse not affiliated with the study if deemed appropriate by BCN-R.

#### The intervention

The intervention and associated materials were developed by the multidisciplinary working party including consumer representatives. The intervention comprised two components.

**Figure 1:**
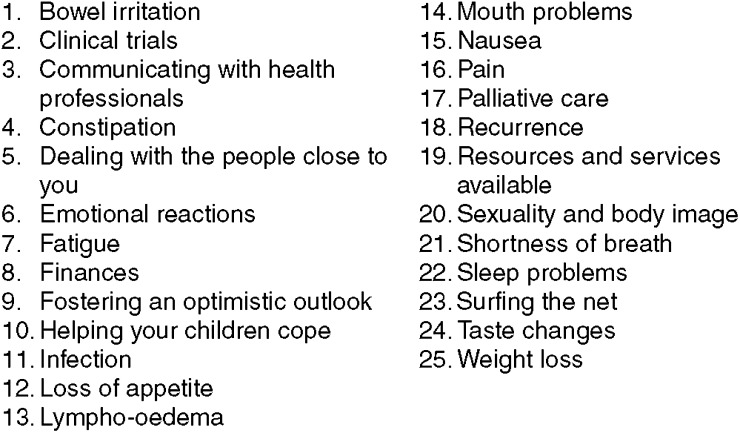
Topics of information cards on self-care and communication strategies.

1. *Intervention Session 1 (face-to-face)* Within 10 days of recruitment, the BCN-R conducted the first intervention session scheduled to last approximately 1 h. Patients were encouraged to bring a significant other. The session comprised four components.
  - *Orientation:* The BCN-R actively listened to patients' concerns, offered empathy and support, and then established the patient's understanding of her situation. Misconceptions were corrected.
  - *Tailored responses:* Information provided was tailored to the woman's individual concerns by asking the woman directly and from responses on baseline questionnaire. Any responses marked ‘high need’ on the SCNS were priorised. The patient identified her top three concerns for discussion.
  - *Coaching and practicing self-care & communication strategies:* After problem definition, coaching and rehearsal of self-care, stress reduction and communication strategies for the problem was provided. The woman's response to strategies was elicited, and then there was discussion of how each strategy might be incorporated into their daily life; finally, a realistic goal was set to achieve in this area.
  - *Concluding the session*: The session was ended by summarising the main issues, reviewing and reinforcing the strategies, asking for further questions, making any necessary referrals and making an appointment time for the follow-up phone call.
  - *Materials and resources*: Twenty-five information cards on self-care and communication strategies were developed. Figure 1 shows the topics. They were based on the best available evidence. The cards were not available to control group. Each woman was given relevant information cards and a copy of her personal self-care plan. Women were also provided with a relaxation CD.
  - *Informing team members*: A written summary of the meeting was provided to the treating doctor and placed in the medical record. The intervention log recorded: when the intervention was delivered, how long the session lasted, the major issues raised; strategies suggested to address these issues and referrals provided.
2. Intervention Session 2 (telephone): The BCN-R telephoned the patient 1 week after the first session to: (a) ask whether the suggested strategies had ameliorated the concerns; (b) elicit and respond to any concerns remaining; (c) reinforce or modify planned self-care strategies or introduce new ones; and (d) prompt for further questions or new concerns.

Other relevant information cards were posted out. Prior to telephoning the woman, the BCN-R reviewed the patient history and first intervention session. The call was scheduled to last for up to 30 min. A summary was provided in writing to the treating doctor. The intervention log was completed. The information included: when the intervention was delivered, the length of the call, and whether the suggested strategies had been used.

#### Training breast care nurses to deliver the intervention

Breast care nurses were recruited from each of the sites (*n*=4). They attended two training days, which covered adherence to the research protocol, and evidence based best-practice medical and psychosocial management of women with advanced breast cancer. A team comprising SA, RF, DM, a medical oncologist, a radiation oncologist and two experienced breast care nurses provided training. Teaching included role plays about difficult situations that may arise. Constructive feedback and debriefing was provided.

#### Follow-up

Follow-up questionnaires comprising EORTC QLQ-30 and SCNS were posted out to all women unless the woman had withdrawn or died. Telephone follow-up was used to enhance response rate. A research assistant, not involved in the intervention delivery, administered all follow-up data collection.

### Measures

#### EORTC QLQ-30 version 2

The quality of life questionnaire contains 30 items and was scored according to the EORTC QLC-30 scoring manual. Subscales include five functional scales, the global health status quality of life scale and nine symptom scales. High scores represent healthy functioning in the functional scales, high quality of life in the global health status quality of life scale and a high level of problems for symptom scales. It has demonstrated high reliability and concurrent and criterion validity (Hjermstad *et al*, 1995; Kaasa *et al*, 1995).

#### Supportive care needs survey

The SCNS contains 59 items designed to measure patients' perceived needs in five core domains: psychologic, health information, physical and daily living, patient care and support, and sexuality. The rating scale is no or satisfied, low, moderate or high need. Items for each subscale were linearly transformed 0–100 and averaged. Higher scores indicate higher level of need. The subscale scores were used in the analyses as the primary outcome. As a secondary outcome, for each subscale score, each woman was categorised as having high baseline needs (a score in excess of 50) or low baseline needs (50 and below). This has high internal consistency and demonstrated construct and content validity (Bonevski *et al*, 2000).

#### Statistical methods

Comparison of baseline characteristics between the usual care and intervention arms used *t*-tests for means and *χ*^2^ tests were used for proportions. Difference scores between 1 and 3 month follow-ups, and baseline EORTC and SCNS scores were calculated for each individual. For EORTC subscales, a negative score indicates that function has decreased and for SCNS subscales, a negative score indicates that needs have decreased. Linear regression was used to evaluate differences in change in EORTC and SCNS scores between the two study arms, adjusting for baseline level of function or need. A *P*-value of <0.05 was considered statistically significant. Outcome analyses were conducted on an intention to treat basis.

## RESULTS

### Patient characteristics

Forty-six patients were randomised to the usual care arm and 59 to the intervention arm. Table 1 shows patient demographic and disease characteristics. On most demographic, disease and treatment variables, patients were similar at entry to the study (all *P*>0.15 for differences between the groups). A greater proportion of patients in the intervention arm were undergoing current radiotherapy (93 *vs* 73%, *P*=0.01). Proportionately more patients in the usual care arm of the study had minor children (38 *vs* 14%, *P*=0.02).

At baseline, EORTC and SCNS mean scores of patients in the usual care arm did not vary significantly from the scores in the intervention arm of the study (all *P*>0.20). Subscale scores were also not significantly different between the arms when classified as the proportion with higher needs or poorer functioning (all *P*>0.13).

### Follow-up

**Figure 2:**
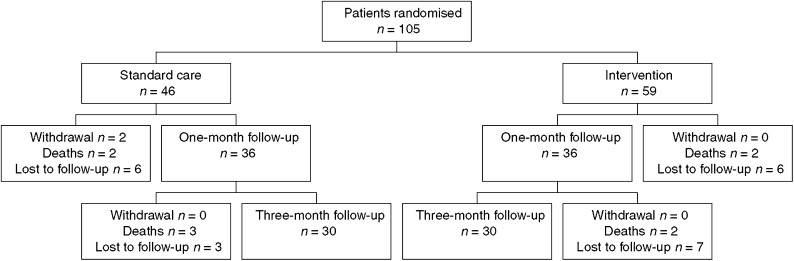
Follow-up by treatment arm.

Overall, 69% (72 out of 105) of patients completed the 1 month follow up, 78% (36 out of 46) in the usual care arm and 61% (36 out of 59) in the intervention arm (Figure 2). Four of the 105 patients (4%) died prior to 1-month follow-up. Follow-up at 3 months was 57% (60/105), 65% (30/46) of usual care patients and 51% (30/59) intervention patients. Four of the 105 patients (9%) died prior to 3-month follow-up. Excluding the deaths, this represents overall response rates of 71 and 63% for the 1 and 3 month follow-ups respectively.

Patients who died, withdrew, or were lost to follow-up in the usual care arm had consistently lower baseline EORTC scores than those who died, withdrew, or were lost to follow-up in the intervention arm (data not shown). However, these groups did not differ at baseline on the SCNS subscales.

### Application of the intervention protocol

#### Intervention session 1 (face-to-face)

Most women (85%) completed the intervention on the date of consent. The remainder (15%) received the intervention between 2 and 10 days later. The time taken for the session ranged from 30 to 100 min (average=59 min). The most common issues discussed were concerns about family (31%), treatment-related issues (31%), fatigue and sleeping difficulties (29%), pain (22%), financial concerns (20%) and loss of independence (13%). The number of issues raised ranged from 0 to 8, with two women raising no issues and most (71%) raising two or three issues. Accordingly, the number of strategies suggested ranged from 0 to 8 with most interviews (66%) resulting in two to three strategies. Twenty-four women were referred to another professional/group. These included support groups (11), social workers (9), medical oncologists (3), dietitian (2) and psychologist (2).

#### Intervention session 2 (telephone)

Twenty per cent of women were unable to be contacted on the time set for contact. All women were ultimately contacted within 2 weeks. Most telephone calls (80%) lasted between 15 and 30 min. The range was 4 to 45 min (average=22 min). Thirty-six per cent of women reported using all the self-care strategies suggested at intervention session 1. The strategies of maintaining a positive outlook, carrying out normal enjoyable activities and having quality ‘me’ time were suggested for anxiety, frustration and the need to feel normal were utilised by 12 women. The strategy offered for family communication difficulties was to encourage open communication and time together to talk about issues but only four out of nine women used this. Fatigue was an issue for 11 women, nine of whom agreed to try a light exercise program in combination with rest but only four women used it.

Four out of the five women who had issues with their oncologists used the strategy of making an appointment to discuss their issues. Two women experienced changes or loss of taste and used the strategy of experimenting with different foods, herbs and spices and found this to be beneficial. Fifty-six per cent of women reported being unable to have all of their needs met.

### Difference between baseline and postintervention EORTC and SCNS domain scores

EORTC physical functioning for both groups tended to increase over baseline level at 1 month by an average of 20–22 points whereas general quality of life decreased on average 26–28 points (Table 2). Other EORTC functional subscale scores on average remained similar to baseline levels in both the usual care and intervention groups. A similar pattern was seen for the 3-month postintervention EORTC domain scores compared to baseline. Adjusting for baseline score, change in EORTC domain scores was not significantly different between the intervention and usual care arms of the study at either 1 month or 3 months postintervention.

A small decrease in SCNS subscale scores was evident 1-month postintervention in the intervention arm of the study (i.e., a decrease in need). In the usual care arm, SCNS domain scores tended to increase slightly. Small decreases in SCNS domain scores were observed in both groups at 3 months postintervention. Adjusting for baseline score, there were no significant differences between the intervention and standard care group for the change in SCNS domain score in any of the domains. However, a nonsignificant trend was observed: the patients in the intervention group had on average a 6-point decrease in scores on the SCNS psychological needs scale compared to a 2-point increase among the standard care patients.

**Figure 3:**
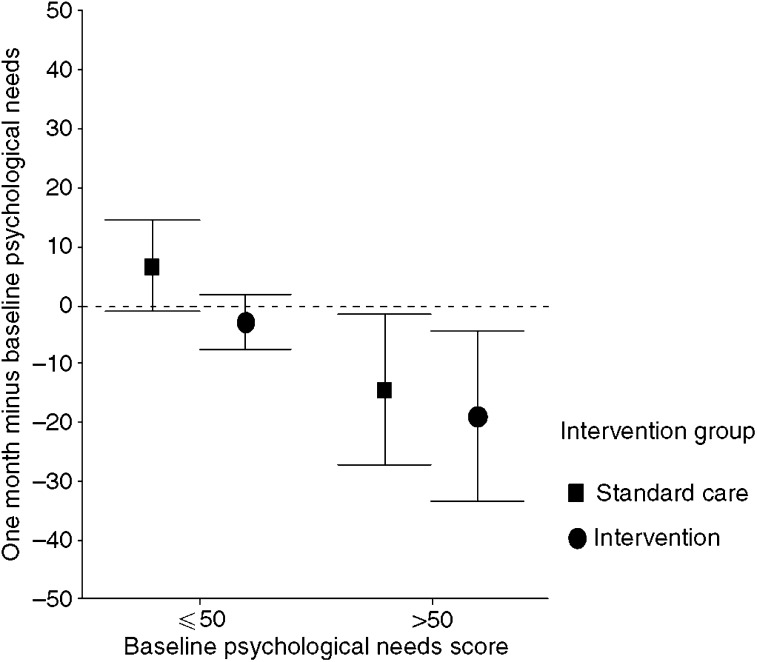
Average change in psychological needs by level of baseline need.

When the sample was stratified by higher (over 50) or lower (50 and below) baseline psychological need score, the intervention group with higher baseline needs reported a 19-point decrease in psychological needs compared to a 14-point difference on average in the usual care group (Figure 3). This difference was significant (*P*=0.026). Among those with lower baseline need, the usual care group on average increased 7 points whereas the intervention group decreased on average 3 points. No significant differences between the intervention and usual care arms were observed at 3 months postintervention on the SCNS domain scales.

## DISCUSSION

Uptake of the intervention was high. Every woman allocated to the intervention group participated in the face-to-face session and the telephone session. The number of concerns that were raised by woman ranged from 0 to 8, with most woman having 2 to 3 concerns. Together, these observations suggest that there is a high level of need among women with advanced breast cancer that is not currently being met by existing standard health care services. The three most commonly raised issues were concerns about family members, treatment-related issues and fatigue. However, the dominant concerns raised by women varied substantially which demonstrates that a one-size fits all approach to meeting patients' needs is inappropriate. Adult learning occurs most effectively when instruction is focused on issues that are highly relevant to the individual at that time (Knowles, 1989).

Overall, the findings did not support the primary hypotheses: there were no significant differences between the two groups in terms of changes in quality of life or unmet needs from baseline. There are a few explanations for the lack of differences. First, perhaps the sample size was not sufficiently large to detect differences between the two groups. A retrospective power calculation suggests that using the sample size obtained, a standardised difference of 0.5 could be detected assuming *P*<0.05 and power of 70%. However, there was no indication of a trend for any differences in change of quality of life between the two groups. For unmet needs subscales, there were nonsignificant trends for differences between the two groups observed at 1 month, but not 3 months. These trends suggested that there might be a drop in unmet needs in the intervention group but not in the usual care group. However, even if the sample size were increased, would these modest differences represent a clinically significant change?

Second, the uptake of the recommended self-care strategies was only moderate. There appeared to be slightly better uptake of psychological/communication strategies than physical strategies. As uptake of the strategies was essential to the success of the intervention in enhancing quality of life and reducing needs, this is clearly a potentially valid explanation.

Third, despite taking a ‘targeted’ approach to the intervention, it may be that the intervention was not sufficiently intense to achieve change. As women's needs vary (Aranda *et al*, 2005), it is clear that systematically assessing the most prominent needs and tailoring the intervention specifically to meeting those needs is an important aspect of an effective intervention. More intervention sessions may well have encouraged greater uptake of the strategies, which in turn may have resulted in clinically significant differences between the two groups. Moreover, 56% of woman said that they did not have all their needs met. More sessions may have allowed a greater number of concerns to be addressed. Northouse *et al* (2005) came to similar conclusions with their trial of a psycho-educational family intervention program that was also based on the FOCUS framework. However, this may create issues for adoption in routine practice as more sessions would be more resource intensive (Schofield *et al*, in press) and patients may not be prepared to attend many more sessions, particularly because fatigue was a major problem (Aranda *et al*, 2005).

There was one important significant effect that indicates that this intervention approach warrants further development. Among woman who expressed high initial psychological needs, those who received the intervention experienced a greater reduction in psychological needs compared with those who did not receive the intervention. This finding reinforces the importance of assessing individual needs and providing this information to the treatment team for intervention, in line with Velikova *et al* (2004) earlier work. It also reflects earlier findings (McArdle *et al*, 1996) that showed that empathy combined with information reduced psychological distress in women with breast cancer. The approach taken with the current intervention was based on cognitive behaviour techniques, which was found to be successful in previous research (Savard *et al*, 2005; Yates *et al*, 2005). It involved actively listening, offering empathy and support, helping patients to identify beliefs contributing to their psychological issues and collaborating with patients to select effective strategies, for example: help patients to restructure negative thoughts to positive thoughts.

Overall, quality of life was observed to slightly decrease over the time of the study. This is not surprising among a group of people with advanced cancer. However, there was a tendency for physical functioning to improve by the 3-month follow-up period. This may be because the clinic treatment teams are identifying and controlling the physical symptoms. Indeed, there is evidence that physical symptoms are more likely to be managed than psychosocial ones. In one UK study of cancer patients, concerns about physical symptoms, which the patients generally rated as of ‘low concern’, were more frequently addressed by the treatment team than the psychological concerns which patients rated as of ‘high concern’ (Hill *et al*, 2003).

## LIMITATIONS

First, the four BCN-Rs reported that 1-week between the intervention and follow-up telephone call was too short, as some strategies involved referrals to other health professionals. Hence, it may be prudent to deliver the subsequent sessions of the intervention at 2-week intervals to allow time for adoption of the care plan to take place. Second, as discussed earlier, more intervention sessions may have produced greater changes in outcomes for the intervention group. Alternatively, a larger sample size may have detected more significant differences between the two groups. Third, the sample comprised urban, English-speaking women. Rural women and women who do not speak English may have different requirements for psycho-educational interventions. Fourth, the consent and retention rate would be generally considered to be low. However, considering this group had metastatic disease and they were being asked to complete three surveys over a 3-month period, the consent rate may be considered reasonable. It has been noted that in studies of people with advanced cancer, recruitment and retention are notoriously difficult (Hudson *et al*, 2001). Nevertheless, there may be some bias in the sample, especially as one of the reasons given for nonconsent was tiredness, which suggests that the effects of the intervention on women with poorer health status may not have been assessed. As no details were collected from the refusers, it was not possible to formally analyse response biases.

## CONCLUSIONS

Despite the fact that the intervention failed to produce global improvements in quality of life and a reduction in perceived needs, this systematic approach to assessing woman's psychological, informational and symptom needs and tailoring the intervention to meeting these needs shows promise. Greater impact on key outcomes may have been achieved if the intervention comprised a greater number of sessions. The finding that women with high psychological needs benefit from a brief intervention suggests the need to undertake intervention-dose testing against level of need in future studies. Further, as uptake of referrals was less than optimal, future interventions should also focus on improving referral uptake as it is critical to the success of the intervention. In conclusion, the work of specialist nurses, such as breast care nurses, is often based solely on experience. This study suggests that as a minimum BCNs should undertake a routine assessment of patient's needs and psychological status. This study also supports the routine incorporation into BCN practice of general skills aimed at eliciting and responding to emotional concerns with women with advanced breast cancer.

## Figures and Tables

**Table 1:** Patient demographic and disease characteristics

	**Standard care**		**Intervention**	
*Age*				
Median (range) years	55 (36–82)		57 (34–85)	
*Years since breast cancer diagnosed*				
Median (range) years	5 (0–26)		5 (0–27)	
*Years since advanced breast cancer diagnosed*				
Median (range) years	1 (0–14)		1 (0–7)	

	***n***	**%**	***n***	**%**
*Clinic*				
Box Hill	7	15	12	20
Cabrini	14	30	15	25
Peter MacCallum	18	39	19	32
The Alfred	7	15	13	22
*Marital status*				
Never married	5	11	6	10
Married/de facto	32	71	35	60
Separated/divorced	6	13	9	16
Widowed	2	4	8	14
*Highest education*				
School certificate	12	28	18	31
Higher school certificate	4	9	7	12
Certificate/diploma	6	14	5	8
University degree/diploma	10	23	13	22
University higher degree	2	5	3	5
Other	9	21	13	22
*Children*				
At least one child <18 years	17	38	8	14
Adult children	21	47	35	61
No children	7	16	14	25
*Current chemotherapy*				
No	18	40	31	55
Yes	27	60	25	45
*Current radiotherapy*				
No	33	73	52	93
Yes	12	27	4	7
*Current hormone therapy*				
No	29	64	30	54
Yes	16	36	26	46
*Current surgery*				
No	44	98	52	93
Yes	1	2	4	7
*Current APD therapy*				
No	23	51	31	55
Yes	22	49	25	45

**Table 2:** Difference in EORTC and SCNS domain scores post intervention adjusted for baseline score

	**Usual care**		**Intervention**	
	**Mean**	**s.d.**	**Mean**	**s.d.**
*Difference between baseline and one month*				
*EORTC functional scales*				
Physical functioning	19.6	23.6	21.7	19.5
Role functioning	−2.8	34.8	−2.0	29.9
Emotional functioning	2.2	24.3	1.7	18.3
Cognitive functioning	1.9	17.3	−2.9	21.3
Social functioning	1.4	29.4	7.8	26.9
General quality of life	−26.2	42.7	−28.1	36.1
*SCNS need scales*				
Psychologic Needs	2.3	21.4	−6.1	17.7
Health information needs	−3.4	21.9	−7.5	27.6
Physical and daily living needs	2.2	19.2	−1.7	14.6
Patient care and support needs	2.2	11.3	0.3	16.7
Sexuality needs	1.3	32.6	−6.5	28.2
*Difference between baseline and 3 months*				
*EORTC functional scales*				
Physical functioning	17.9	23.1	21.6	20.3
Role functioning	1.5	33.9	0.0	32.9
Emotional functioning	5.4	25.6	3.7	20.6
Cognitive functioning	2.0	19.1	0.8	22.4
Social functioning	10.8	29.3	2.4	32.2
General quality of life	−33.6	36.6	−22.6	39.1
*SCNS need scales*				
Psychologic needs	−6.5	21.7	−2.8	18.5
Health information needs	−11.7	25.7	−9.4	23.4
Physical and daily living needs	−3.6	22.6	−2.0	16.4
Patient care and support needs	−4.0	9.4	−1.6	16.2
Sexuality needs	−6.8	25.1	−9.8	28.5
